# Autologous Protein Solution (APS) and Osteoarthritis of the Knee: A Scoping Review of Current Clinical Evidence

**DOI:** 10.7759/cureus.53579

**Published:** 2024-02-04

**Authors:** Ashim Gupta

**Affiliations:** 1 Orthopaedics and Regenerative Medicine, Regenerative Orthopaedics (OPC) Private Limited, Noida, IND; 2 Regenerative Medicine, Future Biologics, Lawrenceville, USA; 3 Regenerative Medicine, BioIntegrate, Lawrenceville, USA; 4 Orthopaedics, South Texas Orthopaedic Research Institute, Laredo, USA

**Keywords:** lr prp, leukocyte-rich platelet-rich plasma, prp, platelet-rich plasma, aps, autologous protein solution, blood-derived biologics, autologous blood, regenerative medicine, knee osteoarthritis

## Abstract

Knees are the most regularly affected weight-bearing joints in osteoarthritis (OA), impacting millions of individuals across the globe. The incidence of knee OA will further rise with increasing rates of obesity and lifespan, resulting in a significant increase in the worldwide socioeconomic burden. Conventional therapies used to manage the symptoms associated with knee OA have limitations. Lately, there has been an increased interest in the use of autologous peripheral blood-derived orthobiologics (APBO), including autologous protein solution (APS), for the management of knee OA. Here, the primary objective is to summarize the outcomes of clinical studies involving APS for the treatment of knee OA. Several databases (Embase, Scopus, PubMed, and Web of Science) were searched using terms for the intervention “APS” and treatment “knee OA” for articles published in English until January 21, 2024. All clinical studies using APS as an intervention for the treatment of knee OA were included. Studies not utilizing APS alone or not aiming at the management of knee OA were excluded. Six clinical studies that met our predefined search terms and inclusion and exclusion criteria were included in this study. The results demonstrated that the intra-articular administration of APS is safe and efficacious in reducing pain and/or improving function in patients suffering from knee OA. However, more multicenter, randomized controlled trials involving active comparators, with adequate power and long-term follow-up along with post-market real-world studies in clinical practice are required to further assess the efficacy of APS and justify its regular clinical use for the management of knee OA.

## Introduction and background

Knee osteoarthritis (OA) is one of the leading causes of chronic disability in individuals because of pain and reduced function, resulting from pathological changes in the articular tissue [1]. It significantly impairs the ADL along with working abilities, leading to a considerable socioeconomic burden worldwide [2]. Conventional treatment modalities for the management of knee OA involve the use of non-pharmacological approaches such as physiotherapy, activity modification and weight management; pharmacological agents such as oral nonsteroidal anti-inflammatory drugs and opioids, and intra-articular (IA) injection of hyaluronic acid (viscosupplementation) (HA) and corticosteroids; nutraceuticals such as glucosamine, chondroitin sulfate, and undenatured type II collagen; minimally invasive procedures such as genicular nerve radiofrequency ablation; and surgical inferences, in progressive stages or after the traditional therapies have been ineffective [1-3]. These abovementioned treatment options have drawbacks and side effects, persistently aiming to decrease pain rather than targeting the underlying etiology [1-3].

Over the last decade, interest in the clinical usage of orthobiologics, including autologous peripheral blood-derived orthobiologics (APBO), has significantly increased for the treatment of knee OA [4]. Platelet-rich plasma (PRP) is the most frequently used APBO [4]. A recent systematic review and meta-analysis assessed 24 randomized controlled trials (RCTs) with 1,344 patients and reported significant improvements in several patient-reported outcome measures (PROMs) including visual analog scale (VAS), knee injury and osteoarthritis outcome score (KOOS), Western Ontario and McMaster Universities Arthritis Index (WOMAC), and International Knee Documentation Committee (IKDC) scores in the PRP group compared to the saline and HA [4]. Nevertheless, the efficacy of PRP remains controversial and is ascribed to a lack of standardized formulation protocol, inter- and intra-patient variables, and so on [5]. Autologous protein solution (APS) is formulated by exposing leukocyte-rich PRP to polyacrylamide beads, to obtain higher levels of growth factors and anti-inflammatory cytokines and lower levels of pro-inflammatory cytokines, thereby compound the anabolic effects of PRP with autologous anti-inflammatory homeostatic characteristics [6]. This study aims to summarize the outcomes of clinical studies involving APS for the treatment of knee OA. The secondary objective is to document the ongoing clinical studies registered on different trial protocol repositories involving APS for the management of knee OA.

## Review

Search criteria

**Figure 1:**
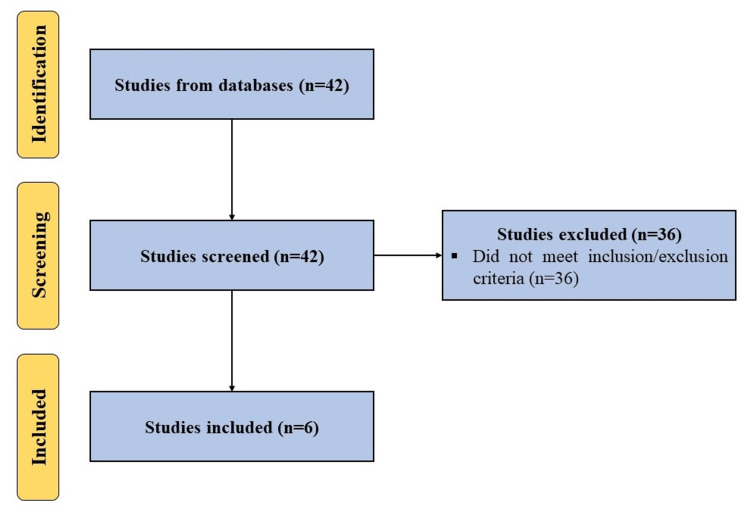
A PRISMA flow diagram outlining the record identification and selection process. PRISMA: Preferred Reporting Items for Systematic Reviews and Meta-Analysis

A search was made using the terms (“autologous protein solution” OR “APS”) AND (“knee osteoarthritis” OR “knee”) in multiple databases including Embase, Scopus, PubMed, and Web of Science for articles published in English until January 21, 2024, while following the Preferred Reporting Items for Systematic Reviews and Meta-Analysis (PRISMA) guidelines. All clinical studies using APS as an intervention for the treatment of knee OA were included. Studies not utilizing APS alone or not aiming at the management of knee OA were excluded (Figure 1). Additionally, we searched Clinical Trials Registry - India (CTRI), ClinicalTrials.gov, and the Chinese Clinical Trial Register (ChiCTR) using the aforesaid search terms to find the listed ongoing clinical trials on the use of APS for the management of knee OA.

Results

Clinical Studies

King et al. [7] in a pilot study investigated the correlation of characteristics of an APS formulation with improvement in the WOMAC score and Outcomes Measures in Rheumatology-Osteoarthritis Research Society International (OMERACT-OARSI) responder status post-administration of APS. Eleven subjects (four females and seven males) were enrolled in this study. APS was formulated according to the manufacturer’s instructions (nSTRIDE APS kit, Zimmer Biomet, Warsaw, IN). WOMAC scores were measured at baseline and one week, two weeks', four weeks', three months', and six months' follow-up. OMERACT-OARSI criteria were used to determine the responder status post-IA administration of APS at the aforesaid follow-up visits. WBC and cytokine concentrations were also measured from the formulated APS and whole blood samples. The APS formulation showed significantly higher concentrations of anti-inflammatory cytokines including interleukin (IL)-1 receptor antagonist (IL-1RA). WBC concentration in the APS and whole blood was also positively correlated (statistically significant) with IL-1RA in APS. The ratio of IL-1RA to IL-1β in the whole blood and APS were significantly correlated with improved WOMAC pain scores at one week and six months' follow-up. Both WBC and IL-1RA concentrations in APS were significantly correlated with improved WOMAC pain scores at one week's follow-up. Additionally, IL-1RA concentration in whole blood was significantly correlated with improved WOMAC pain scores at six months' follow-up. Additionally, 85.7% of the subjects whose IL-1RA to IL-1β ratio was greater than 1,000 or a WBC count >30,000/µL were OMERACT-OARSI responders at six months' follow-up. These results demonstrated that WBC concentration and ratio of IL-IRA to IL-1β are potential developmental candidates to forecast the efficacy of APS in specific individuals. This needs to be further investigated, and the foundation must be laid for future studies to determine the safety and efficacy of APS in knee OA patients.

Drumpt et al. [8] investigated the safety and efficacy of single APS injection in knee OA patients. Eleven participants with unilateral knee OA (Grade II or III on the Kellgren-Lawrence (KL) scale) were included in this study. Adverse events and PROMs including WOMAC and the subject’s and physician’s assessment of global change in the index knee were evaluated at one week's, two weeks', one month's, three months', and six months' follow-up. Long-term follow-up was also performed at 18 months' follow-up, and subjects were assessed on all WOMAC subscale scores and OMERACT-OARSI high pain responder status. APS was formulated per the manufacturer’s instructions (nSTRIDE APS kit, Zimmer Biomet, Warsaw, IN). No adverse effects were reported throughout the study. Both subjects and physicians rated considerable improvements in global assessment of OA severity, with over 80% of the subjects reporting it as “much improved” or “very much improved” at three months' and six months' follow-up. The WOMAC composite score, WOMAC pain score, and WOMAC stiffness and function subscale scores were significantly improved by two weeks, continued to improve by three months, and remained stable by six months' follow-up. OMERACT-OARSI responders' mean WOMAC pain score also showed significant improvement by two weeks' follow-up compared to the baseline. In terms of long-term follow-up, the subjects (n=6) reported significant improvements in all WOMAC subscale scores at 18 months' follow-up, and five out of six included patients met the OMERACT-OARSI high pain responder status. The results demonstrated that IA administration of APS is safe and has the potential to mitigate symptoms associated with knee OA. This study laid the foundation for future, adequately powered, multicenter RCTs to further establish the efficacy of APS in knee OA patients.

Kon et al. [9] in a multicenter, randomized, double-blind, saline-controlled study investigated the efficacy of a single IA injection of APS in reducing pain and improving function in patients with knee OA. Approximately 46 patients with unilateral knee OA, Grade II or III on the KL scale, were randomized 2:1 into the APS (n=31) and saline group (n=15). PROMs including VAS, KOOS, Short Form-36 (SF-36), Clinical Global Impression of Severity/Change (CGI-S/C), Patient Global Impression of Severity/Change (PGI-S/C), and OMERACT-OARSI responder rate were recorded at baseline and two weeks and one, three, six, and 12 months' follow-up. Structural changes via radiographs and magnetic resonance imaging (MRI) were also assessed from baseline to 12 months, and at three months and 12 months post-injection, respectively. APS was formulated per the manufacturer’s instructions (nSTRIDE APS kit, Zimmer Biomet, Warsaw, IN). Cytokine analysis showed high levels of anti-inflammatory cytokines and low levels of pro-inflammatory cytokines in the formulated APS. No major adverse effects related to APS were reported throughout the study. For WOMAC, no significant differences were reported for both APS and saline groups at two weeks and one, three, and six months' follow-up compared to the baseline. However, at 12 months' follow-up, both groups showed significant improvement compared to the baseline, and the improvement in the APS group was significantly higher than the saline group. Additionally, the APS group showed significant improvement in the SF-36 bodily pain subscale, SF-36 role emotional health subscale, and CGI-S/C compared to the saline group at 12 months' follow-up. Additionally, an MRI analysis showed significant improvement in terms of bone marrow lesion size and osteophytes in the central zone of the lateral femoral condyle in the APS group compared to the saline group. No significant improvement for VAS, KOOS, responder rate, and radiographic analysis was observed between the groups at any follow-up time points. The results from this study demonstrated that the administration of APS is safe and led to clinical improvements in patients suffering from knee OA.

Kon et al. [10] in a subsequent study, followed the patients from the previous study [9] to a longer follow-up of 36 months. Patients in the saline group could crossover at 12 months' follow-up. Patients were reassessed at 24 months and 36 months' follow-up for VAS, WOMAC, KOOS, SF-36, and OMERACT-OARSI responder rate along with the radiographs and MRI. No major adverse effects related to the APS administration were reported throughout the study. In the APS cohort, WOMAC pain, WOMAC stiffness and function, VAS, and KOOS showed significant improvement at 36 months' follow-up compared to the baseline. However, the improvement in the VAS at 36 months was significantly lower than the 12 months' follow-up. All patients in the saline group crossed over to the APS group at 12 months' follow-up. These patients showed significant improvement in VAS, WOMAC pain, function and stiffness, and KOOS pain, symptoms, QoL, and ADL at 36 months' follow-up compared to the original baseline (preinjection). The number of responders also increased from seven at 12 months to nine at 24 months and 36 months' follow-up. Additionally, the physical component of SF-36 also improved significantly at 36 months compared to the baseline. No significant differences were observed on radiographs and MRI analysis; however, patients without full cartilage loss at baseline showed significant improvement in WOMAC pain, even if their WOMAC pain at baseline was worse. The results from this study demonstrated that the administration of APS is safe and efficacious in patients with mild to moderate knee OA.

Genechten et al. [11] investigated the advantages, durability, and safety of APS injection in a middle-aged female-only cohort suffering primarily from unilateral patellofemoral OA. The patients were assessed at baseline and one, three, and six and 12 months' follow-up and PROMs measured included numeric pain rating scale (NPRS), KOOS, Kujala anterior knee pain symptom questionnaire, University of California at Los Angeles (UCLA) activity score, and European Quality of Life 5 Dimensions (EQ-5D) VAS. Subjects who showed improvement of >10 points on the KOOS pain subscale were considered responders. A second injection of APS was permitted after three months if the subject deemed the clinical response to be insufficient. Fifty subjects (mainly moderate to severe OA) were enrolled in the study and 41/50 subjects completed the 12 months' follow-up study assessments. APS was formulated per the manufacturer’s instructions (nSTRIDE APS kit, Zimmer Biomet, Warsaw, IN). Statistically significant improvements were observed for NPRS, KOOS, and Kujala scores at 12 months' follow-up compared to the baseline. No significant differences were observed for UCLA and EQ-5D VAS scores. 28/50 enrolled subjects received a second injection. 10/27 of these subjects were considered responders at three months' follow-up and after the second dose of APS, the responder rate increased to 14/27 subjects at six months and 11/23 subjects at 12 months' follow-up. On the other hand, the responder rate in the single injection cohort was 12/19 subjects at six months' follow-up and 11/18 subjects at 12 months' follow-up. The overall patient responder rate in the study was 53.7%. The MRI analysis showed that patients with major synovitis showed significant improvement in KOOS symptoms, KOOS ADL, and Kujala score at 12 months' follow-up compared to the patients with non-synovitis. The results from this study demonstrated over a 53% responder rate in the middle-aged female population. Major synovitis seemed to be a favorable forecaster for pain relief and functional improvement. Temporary adverse effects were reported, but they did not affect the clinical outcomes. 

Kuwasawa et al. [12] in a retrospective study investigated the efficacy of APS in clinical practice in KL grade II to IV knee OA patients. Approximately 220 knees (KL II: 80, KL III: 74, KL III: 66) were enrolled in this study. PROMs included KOOS and OMERACT-OARSI and were assessed at baseline and one, three, six, and 12 months' follow-up. APS was formulated per the manufacturer’s instructions (nSTRIDE APS kit, Zimmer Biomet, Warsaw, IN). Seventy-two knees were lost to follow-up by 12 months, leaving 64, 54, and 30 knees in KL grades II, III, and IV, respectively. The follow-up rate in KL grade IV was significantly lower than in KL grades II and III at 12 months' follow-up. No major adverse events related to the administration of APS were reported. All KOOS subscales showed significant improvement at 12 months' follow-up compared to the baseline. The subgroup analysis showed that patients in KL grades II and III showed significant improvement in a step-by-step manner up to three months' follow-up, and it was maintained until 12 months' follow-up. However, patients in KL grade IV showed similar levels of improvement at three and six months' follow-up, but the improvement declined afterward until 12 months' follow-up, and there was no significant difference in the KOOS at 12 months' follow-up compared to the baseline. Additionally, at 12 months' follow-up, improvement in KOOS in KL grade II was significantly higher than in KL grade IV. The responder rate in KL grade IV was lower than in KL grade II or III. The results from this study demonstrated that the administration of APS is safe, and clinical outcomes in severe cases were inferior to mild-to-moderate cases of knee OA.

The results from the abovementioned clinical studies are summarized in Table 1.

**Table 1:** Summary of main findings of the included clinical studies. WBCs: white blood cells; IL-1β: interleukin-1β; IL-1RA: interleukin-1 receptor antagonist; WOMAC: Western Ontario and McMaster Universities Arthritis Index; APS: autologous protein solution; OMERACT-OARSI: Outcomes Measures in Rheumatology-Osteoarthritis Research Society International; IA: intra-articular; CGI-S/C: Clinical Global Impression of severity/change; KOOS: knee injury and osteoarthritis outcome score; NPRS: numeric pain rating scale; MRI: magnetic resonance imaging; KL: Kellgren-Lawrence.

Author [Reference]	Main Findings
King et al. [7]	This study demonstrated a positive correlation between the concentration of WBCs and ratio of anti-inflammatory IL-1RA to pro-inflammatory IL-1β in the APS formulation and the WOMAC pain score. Additionally, 85.7% of the subjects whose IL-1RA to IL-1β ratio was greater than 1,000 or a WBC count >30,000/µl were OMERACT-OARSI responders at six months' follow-up.
Drumpt et al. [8]	Administration of IA APS is safe and led to significant improvement in all WOMAC subscales and attainment of OMERACT-OARSI high pain responder status at 18 months ' follow-up.
Kon et al. [9]	Administration of IA APS is safe and led to significant improvement in WOMAC, SF-36 bodily pain subscale, SF-36 role emotional health subscale, and CGI-S/C scores compared to the saline group at 12 months' follow-up.
Kon et al. [10]	Administration of IA APS is safe and led to significant improvement in VAS, all KOOS and WOMAC subscales, and SF-36 physical component compared to the baseline at 36 months' follow-up. The number of responders also increased from seven at 12 months' follow-up to nine at 24 and 36 months' follow-up.
Genechten et al. [11]	Administration of IA APS led to significant improvement in NPRS, KOOS, and Kujala scores at 12 months' follow-up. Additionally, MRI analysis showed significant improvement in KOOS symptoms and ADL subscales, and Kujala score in patients with major synovitis compared to patients with non-synovitis at 12 months' follow-up. Additionally, an overall responder rate of 53.7% was observed.
Kuwasawa et al. [12]	Administration of IA APS is safe and led to significant improvement in KOOS subscales compared to the baseline at 12 months' follow-up. The subgroup analysis showed that improvement in severe grades, that is, grade IV (on KL grade), was inferior to mild-to-moderate grades (grade II or III). The improvement in KOOS for KL grade II was significantly higher than in KL grade IV at 12 months' follow-up.

Ongoing Clinical Studies

As of January 21, 2024, there is only one clinical trial registered on CTRI, ClinicalTrials.gov, or ChiCTR to study the safety and/or efficacy of APS for knee OA treatment. This trial is summarized in Table 2. 

**Table 2:** Ongoing clinical trial registered on the Clinical Trials Registry - India, ClinicalTrials.gov, and Chinese Clinical Trial Register until January 21, 2024, evaluating the safety and/or efficacy of APS for the management of knee osteoarthritis.

Study Identifier	Biologic	Study Phase; Estimated Enrollment (N)	Primary Outcome Measure(s)	Recruitment Status	Study Location(s)
NCT03182374	APS vs. hylauronic acid	Not applicable; N=246	Comparing autologous protein solution (APS) with hyaluronic acid (HA) intra-articular injections - Western Ontario and McMaster Universities Osteoarthritis Index (WOMAC) LK 3.1 pain score (time frame: 12 months).	Unknown	Belgium, Denmark, Germany, Italy, Netherlands, Norway, Spain, Switzerland, Turkey, United Kingdom

Discussion

Primary OA, more prevalent than secondary OA, is associated with a detrimental inflammatory cycle, led by pro-inflammatory cytokines, including IL-1β and tumor necrosis factor-alpha (TNF-α) [13,14]. Pro-inflammatory cytokines play a vital role in cartilage matrix degradation via increased production of matrix metalloproteinases. This results in the initiation of the inflammatory response, which triggers a positive feedback loop involving inflammatory cytokines-induced tissue damage, leading to further production of inflammatory cytokines [13,14]. This vicious cycle results in continued degradation of cartilage, causing advanced OA [13,14]. APS is formulated to manage OA by targeting the inflammatory pathways mediated by IL-1β and TNF-α. Previous studies involving the analysis of APS formulation have identified high concentrations of anti-inflammatory and anabolic cytokines and low concentrations of catabolic cytokines [14,15]. Moreover, it is reported that APS formulated from the whole blood of OA patients has high levels of WBCs, platelets, and anti-inflammatory cytokines [6]. Thus, IA administration of a formulation with high concentrations of anti-inflammatory and anabolic cytokines may have the potential to protect or prevent the progression of OA and improve OA-associated symptoms.

In the present study, we reviewed the therapeutic potential of APS for the management of knee OA. Clinical studies aiming at the efficacy of APS for the treatment of knee OA were included. Based on our predefined search and inclusion and exclusion criteria, six clinical studies fit the scope of our review.

A study by King et al. [7] demonstrated a positive correlation between the concentration of WBCs and the ratio of anti-inflammatory IL-1RA to pro-inflammatory IL-1β and the WOMAC pain score, highlighting the importance of these factors in forecasting the efficacy of APS formulation. Recent prospective clinical studies, with follow-up ranging from 12 to 36 months, demonstrated that administration of APS is safe, led to significant improvement in various PROMs measuring pain and/or function, and attainment of considerable responder rate in patients with symptomatic knee OA [8-11]. Additionally, a retrospective study by Kuwasawa et al. [12] conducted in a real-world clinical setting demonstrated that IA injection of APS is safe and efficacious in mild-to-moderate knee OA patients. This study [12] helped integrate the evidence from real-world populations with differing characteristics with prospective clinical studies [7-11], where the conditions are controlled, thereby improving the wholeness of evidence-based medicine evaluations [16]. One ongoing clinical trial is registered on the clinical trial protocol repositories (Table 2).

## Conclusions

In conclusion, despite limitations such as the lack of active comparators, the results from the aforementioned studies demonstrated that IA injection of APS is safe and efficacious in terms of reducing pain and/or improving function in patients suffering from knee OA. However, more multicenter, randomized controlled trials involving active comparators, including other APBOs such as PRP, with adequate power and long-term follow-up along with post-market real-world studies in clinical practice are required to further assess the efficacy of APS and justify its regular clinical use for the management of knee OA.
